# Dementia Care Competency Model for Higher Education: A Pilot Study

**DOI:** 10.3390/ijerph20043173

**Published:** 2023-02-11

**Authors:** Jayne Josephsen, Kirk Ketelsen, Melody Weaver, Hanna Scheuffele

**Affiliations:** 1 School of Nursing, Boise State University, 1910 W University Drive, Boise, ID 83725, USA; 2 School of Public and Population Health, Boise State University, 1910 W University Drive, Boise, ID 83725, USA; 3 School of Nursing, Graduate Studies, Idaho State University, 921 S 8th Ave, Pocatello, ID 83209, USA; 4 Idaho Caregiver Alliance, Center for The Study of Aging, Boise State University, 1910 W University Drive, Boise, ID 83725, USA

**Keywords:** Alzheimer’s disease and related dementias (ADRD), dementia care, competency model, higher education

## Abstract

A statewide landscape analysis was initiated to identify workforce development and educational needs concerning the support of persons with Alzheimer’s Disease and Related Dementias (ADRD). Educational programs preparing healthcare professionals were targeted since people with ADRD, and their families/caregivers, often have frequent, ongoing contact with healthcare providers. A literature review and thematic analysis discovered a dearth of research and a lack of consistent competency identification for healthcare education. A crosswalk comparison of various competency models led to the development of a five factor model. A survey based on this model was sent to educators statewide evaluating confidence in ADRD-specific competency attainment in graduates. Descriptive statistics and factor analysis led to a revision of the original five factor model to a three factor model, including competencies in Global Dementia knowledge, Communication, and Safety, each with various sub-competencies. Identifying ADRD-specific competencies for graduating healthcare students is essential. This three factor competency framework will support educational programs in examining curricular offerings and increasing awareness concerning the needs of the ADRD population. Furthermore, using a robust competency model for healthcare education can assist in preparing graduates to address the needs of those with ADRD as well as the needs of the family/caregiving system and environment.

## 1. Introduction

The Alzheimer’s Association and the Centers for Disease Control and Prevention have developed the Third Healthy Brain Initiative Road Map. The current Road Map emphasizes awareness and understanding of brain health, supporting those with Alzheimer’s Disease and Related Dementias (ADRD), and identifying actionable steps to meet this goal. A vital component of the road map is to ensure a competent workforce, including a specific objective of strengthening the competencies of providers who deliver care to those with ADRD [1].

An estimated 6.5 million people over age 65 live with Alzheimer’s disease. By 2060 it is projected that those over age 65 with Alzheimer’s disease will reach 13.8 million people in the United States, an increase of approximately 47% [2]. Although there are treatments for ADRD, there is no cure, and the disease can progress over long periods. For the post-dementia diagnosis, the average length of life is 4 to 8 years, with some people living as long as 20 years [1] (p. 8). Due to the length and progressive nature of the disease, people with ADRD and their families/caregivers often have frequent, ongoing contact with healthcare providers. The staggering statistics related to the number of people affected by ADRD and the advancement of brain health initiatives in the United States reveal the substantial need for a trained and prepared workforce to care for those with ADRD, their families, and their caregivers. 

A statewide landscape analysis and needs-based assessment project were initiated to align with the Healthy Brain Initiative Road Map and provide information on strengths, gaps, and resources needed to improve one state’s public health response and support for those with ADRD and their families/caregivers. This project was funded by the state ADRD program utilizing state dollars designated by the Legislature. Workforce development and needs were areas of interest, as there were minimal regulations and no statewide curriculum or certification available to healthcare providers. Additionally, a key goal for the public health response was to expand educational opportunities, support, and services for those with ADRD. 

Educators in higher education healthcare-related programs were surveyed to meet this aspect of the landscape analysis. Surveying educators was deemed essential to identifying the current landscape of program offerings, as educators in higher education have an integral role in creating and implementing the curriculum. The purpose of the survey was to understand the perceived readiness of students to provide dementia-specific care, identify gaps or barriers to integrating dementia-specific care competencies into curricula, and outline core dementia care competencies needed at graduation. 

## 2. Literature Review

Many providers across the healthcare workforce interact with those affected by ADRD, including licensed and unlicensed/direct-care personnel. Dementia care is no longer limited to specialist clinics or memory care units but may present across all healthcare areas [3]. Lack of effective communication, lack of identification of unmet needs, and lack of person-centered care practices can contribute to untoward outcomes, such as extended hospital stays or lower quality of life [4]; this creates a need for dementia-specific training in educational programs. 

Necessary training related to dementia-specific care for providers in health care is inconsistent throughout the United States. Training requirements vary by state or licensure, with few states requiring specific dementia training in their statutes or regulations [5]. Dementia-specific training has primarily focused on direct care personnel, as they often provide most of the “hands-on” care for those with ADRD [6]. Training is often “on the job” or offered as a continuing education requirement for licensure rather than training provided in educational programming in preparation for practice post-graduation [3,6,7,8].

In states with post-graduation or licensure-specific dementia-related training requirements, the training usually includes competencies in communication, the unique needs of those with dementia, understanding and responding to behavioral symptoms, and techniques to address the effects of dementia [5]. Other common content areas in organizationally designed and delivered dementia training may include dementia risk reduction, assessment and diagnosis, pharmacological interventions, and end-of-life care [9]. As with required training for licensure or practice, defining the core competencies of dementia-specific care is inconsistent. It may focus on a specific profession or “direct” interaction with the person living with dementia.

Fazio et al. (2018) outline dementia care practice recommendations for professional healthcare providers: person-centered care, detection and diagnosis, assessment and care planning, medical management, information, education and support, ongoing care for symptoms, support for activities of daily living, staffing, supportive and therapeutic environments, and transitions and coordination of services [10] (p. S1). Callahan et al. (2014) present a best practice model with a foundational principle of the “recipient-caregiver dyad” [11] (p. 4). Warshaw and Bragg (2014) suggest that healthcare curricula planners incorporate competencies in geriatrics, gerontology, dementia care, and team-based care [12] (p. 639). The presence of several different frameworks concerning dementia care competencies makes it difficult for educational programs to identify the foundational knowledge, skills, attitudes, and competencies students need to possess at graduation in order to provide relevant and competent care to those with ADRD and their families/caregivers.

## 3. Materials and Methods

Educational preparation of the workforce was identified as a critical component of the state landscape analysis and needs-based assessment; a literature review was conducted to determine a dementia care competency framework that could be applied to various educational programs and curricula. Upon review of the literature, there appeared to be a lack of research on dementia-care-specific competencies at a curriculum level in higher education. The need to review a variety of dementia-specific competency frameworks and to develop one that could be applied to educational programming was identified as a critical component of the educator survey development. An integrative review of the literature was performed to gather and synthesize various dementia care competency frameworks. Multiple sources and established professional guidelines were reviewed to identify common and overlapping themes of core competencies across disciplines and settings [13]. 

A modified Braun and Clark (2006) thematic analysis technique was utilized to identify recurring themes in the literature and understand dementia care competencies across disciplines [14]. Reviewing online databases and performing internet searches to find national, state, local, and organizational frameworks assisted with familiarization with data concerning dementia care competencies. The online databases used to obtain literature were: Academic Search Premier, CINAHL with full text, Health Source: Nursing and Academic edition, and MEDLINE. Terms used were Dementia AND Competencies, Dementia AND Framework, and Dementia AND Skills. Articles up to 10 years old were reviewed. The same search terms were used for the internet literature search. Questions directing the thematic analysis of resources included: “What dementia care competencies are seen throughout disciplines?”, “What are priority dementia care goals?” and “What does quality dementia care consist of?”. Initial codes generated included dementia knowledge, communication, person-centered care, well-being, caregivers, and interdisciplinary care. Themes were created and reviewed by creating a competency crosswalk instead of generating a thematic map, as in the Braun and Clark (2006) model [14]. 

The competency crosswalk was selected, rather than a thematic map, since competency identification is foundational to curriculum development, and competency attainment is essential for accreditation in higher education. Identification of competencies reflects student expectations and learning outcomes across programs [15]. Constructing a competency crosswalk allowed for the analysis of overlapping themes, relationships, and connections. Crosswalk construction moved stepwise from exploratory to confirmatory, examining various resources before determining which competency frameworks to include in the crosswalk development. Four dementia care competency frameworks were selected to provide the foundation for thematic analysis and the initial dementia care competency model development. The frameworks selected represented licensed/professional and unlicensed/direct care workforces. The established frameworks represented professional journals, a national agency supporting community care, and a framework developed at the state level. These frameworks were selected as they represented a variety of perspectives in dementia-care-specific competencies and would ideally translate across various educational programs, settings, and disciplines (See Appendix A for The Dementia Care Competencies for Educational Programs Crosswalk). 

Once the competency crosswalk was developed, each theme was reviewed, defined, and named to create clear competencies paired with student outcomes. Five dementia care competency areas were identified: knowledge of dementia, communication/interaction with persons with dementia, person-centered care, interdisciplinary care, and care for self and caregivers. The student outcomes were edited for clarity and context and paired with the dementia care competencies. This framework was reviewed and assessed for ease of readability and translation into a survey format to be delivered to educators. A series of 15 questions from the five competency areas was created to assess educator confidence in the skills/knowledge of graduates related to dementia care. Possible responses to the survey included: don’t know, not confident, slightly confident, moderately confident, and very confident (See Appendix B for the Educator Survey with Dementia Competency Pairing). The final drafted survey was submitted to the University Office of Research Compliance and received IRB approval (IRB #: 186-SB22-011).

Distribution of the survey was completed through purposive convenience sampling and the use of the Qualtrics© system [Provo, UT, USA]. Potential participants were identified by reviewing faculty directories from departments and programs in higher education institutions across the state. Survey invitations were then sent to faculty and administrators whose students would likely have future interactions with patients, families, or community agencies involving dementia care. Participants were invited to share the survey with additional faculty or administrators, and additional invitations were sent directly to selected individuals. 

## 4. Results

Approximately 485 persons were invited to participate, with 75 responses (a response rate of 15%). The final sample (*n* = 70) consisted of Administrators (*n* = 17), Faculty (*n* = 46), Adjunct Faculty (*n* = 4), and Other (*n* = 3). Seventy-four percent reported five or more years in higher education. Prior to analyses, frequencies were run to assess data accuracy, identify potential outliers (of which there were none), and identify any missing data and/or patterns of missingness. Six of the seventy participants did not take the survey and were listwise deleted from subsequent analyses. Descriptive statistics for the 15 items can be seen in Table 1.

A Factor Analysis using Generalized Least Squares (GLS) with Promax rotation was used in an initial run to estimate the likely number of dementia care competency factors using eigenvalues. The recommended methodology for factor analysis of ordinal data with less than five categories (and when the response distribution may be non-normal) is generally least squares [16]. Using eigenvalues greater than 1 for the initial solution yielded a 3 factor structure, with the first factor (eigenvalue = 8.18) accounting for 54.5 percent of the variance. Two subsequent factors having eigenvalues greater than 1 accounted for 7.9 and 7.4 percent of the variance, respectively. Several additional runs were performed to arrive at a final solution. In an effort to establish some evidence of reliability, Cronbach’s alpha was run on the 15 items. The alpha for the overall scale was relatively high (*r* = 0.939). None of the items would improve the alpha if removed. The inter-item correlation matrix and rotated factor loadings are presented in Table 2. All analyses were run using JMP(v16) [Cary, NC, USA]. 

Several criteria were used to determine the best model (i.e., the number of factors). Using Cudeck and Henly’s (1991) framework utilizes simple fit indexes and χ^2^ [17]. Root mean square error of approximation (RMSEA) [18,19] for the one, two, and three factor models was assessed. RMSEA is considered an appropriate measure when the goal is to maximize verisimilitude [20]. While none of the models dropped below popular guidelines (RMSEA ≤ 0.06) recommended by Hu and Bentler (1999), the 3 factor model had the smallest RMSEA (0.138) [21]. Akaike’s information criteria (AIC) [22] is another popular index used to assess model fit. Again, the 3 factor model had the lowest AIC (19.577) compared to the 2 and 1 factor models (54.999 and 96.997, respectively); therefore, the 3 factor model was retained. 

Factor analysis results directed the revision of the original five dementia care competency frameworks to one with three overarching competencies, categorized as Global Dementia Knowledge, Communication, and Safety, each with various sub-competencies. The factor analysis suggested a positive correlation in global dementia knowledge, indicating the need for graduating students to have a broad global understanding of dementia and how to plan and coordinate care for those with dementia. The second factor detected was communication, signifying the need for the graduating student to possess various communication skills that can cross settings and are situationally adaptable. The third factor distinguished was safety, revealing the need for graduating students to have a breadth of knowledge concerning potential adverse outcomes in the care of those with ADRD and the need to have situational awareness. See Table 3 for the revised three factor dementia care competency model with sub-competencies.

## 5. Discussion

This pilot was completed to outline core dementia-specific care competencies and gaps in training for healthcare providers interacting with those with ADRD, their families, and their caregivers. Through the iterative process of factor analysis, it became clear to the researchers that the initial assumption of the five factor model was no longer evident. Results highlighted the need for graduating students to have a broad knowledge of dementia, understand how to communicate across settings, and safely navigate various situations effectively. The three factor model of Global Dementia Knowledge, Communication, and Safety deviates from the initial five competency model but may categorize dementia-specific care competencies more efficiently and effectively.

As a pilot study often uncovers areas for further development, this three factor model of Global Dementia Knowledge, Communication, and Safety indicates the need for further expansion of survey questions to address aspects of Communication and Safety. The subsequent research phase will assess higher education faculty’s confidence in teaching components of the dementia care competency model. This will include adding more questions to address the weakest factors identified. This pilot has proven to be an informative first step in quantifying the competencies needed for dementia-specific care. It is recommended that this pilot be restructured to the three factor model and tested on a larger population, continuing to refine the categories and subcategories. This will assist in defining the three factor model in a manner that is valid and generalizable for use in various higher education institutions. 

## 6. Conclusions

Educational programs are vital to reframing how brain health and ADRD are understood by providers who care for those with ADRD and their families/caregivers. A workforce trained in dementia-specific competencies is essential to advance all aspects of the Healthy Brain Initiative Road Map and provide relevant and person-centered care for those with ADRD and their families/caregivers. The knowledge, skills, and attitudes needed to engage in person-centric care planning, engagement, and decision-making are foundational and, appropriately, would be a core component of healthcare-related curricula. Developing core dementia care competencies is essential to advance how healthcare professionals are prepared in their educational programs. Through the development of this pilot dementia care competency model and survey, educational programs can examine curricular offerings, increasing awareness concerning the needs of the ADRD population and the value of including relevant dementia care content in the curriculum. The three factor dementia care competency model of Global Dementia Knowledge, Communication, and Safety, along with their constituent sub-competencies, can guide future curriculum development and integrate dementia care competencies into current curricula. These competencies can be incorporated into various educational programs, including certificate, undergraduate, and graduate curricula [7,23]. 

Limitations of the proposed three factor model of dementia care competencies relate to the breadth of survey respondents. The survey was only delivered in one state and had a 15% response rate. The proposed three factor model of Global Dementia Knowledge, Communication, and Safety competencies should be the basis of a revised survey to identify if the proposed three factor model is supported through various educational programs and disciplines, as well as regions and states. Future research across disciplines, institutions, and geographical areas will ensure that the three factor dementia care competency framework addresses the educational and workforce development needs of those working with the ADRD population. Additionally, further research may be indicated to ascertain if the competency framework might also address the educational and training needs of those who work with other chronic degenerative disease populations. 

This study provides evidence for a three factor model of dementia care competencies, including Global Dementia Knowledge, Communication, and Safety, along with relevant sub-competencies. Identifying critical competencies for graduating students who will work with and care for those with ADRD is essential for appropriate workforce development that will meet the needs of our communities and those with ADRD and their families/caregivers. Identifying the central dementia care competencies and sub-competencies will assist higher education programs in preparing those working with and caring for those with ADRD, examining current curricular offerings, and identifying gaps in graduate practice preparation. 

## Figures and Tables

**Table 1:** Dementia Competencies Educator Survey Descriptive Statistics (*n* = 63).

How Confident Are You That Graduates of Your Program have the Ability to:	Mean (1 to 5 Scale)	SD
Describe, in lay language, the progression of dementia	2.75	0.842
2. Identify cognitive and non-cognitive symptoms of dementia	2.86	0.895
3. Differentiate dementia from delirium or depression	2.68	1.045
4. Adapt verbal communication strategies to meet the needs of the person living with dementia	2.76	0.856
5. Adapt non-verbal communication strategies to meet the needs of the person living with dementia	2.65	0.864
6. Develop a person-centered plan of care for persons living with dementia	2.56	0.963
7. Recognize the importance of cultural background and lived experience of the person living with dementia when developing a plan of care	2.62	0.974
8. Analyze the ethical and legal parameters of providing care for a person living with dementia	2.37	0.903
9. Incorporate support of the family caregiver into the plan of care for a person living with dementia	2.54	1.013
10. Recognize signs and symptoms of caregiver stress and burnout	2.60	1.040
11. Assist the caregiver in identifying available resources and services	2.54	0.981
12. Communicate with a multi-disciplinary team of health and social service providers when caring for a person living with dementia	2.98	0.852
13. Identify signs of possible self-neglect, abuse, or exploitation in a person living with dementia	2.89	0.918
14. Identify signs of possible self-neglect, neglect, abuse, or exploitation in a caregiver providing care for a person living with dementia	2.67	1.000
15. Identify ways to promote your personal safety when working with persons living with dementia	2.81	0.840

**Table 2:** Survey Inter-Item Correlation Matrix and Rotated Factor Solution *.

How Confident are you that Graduates of your Program have the Ability to:	Factor 1	Factor 2	Factor 3
Incorporate support for the family caregiver into the plan of care for a person living with dementia	0.834	0.103	0.015
2. Recognize signs and symptoms of caregiver stress and burnout	0.791	−0.086	0.226
3. Develop a person-centered plan of care for person living with dementia	0.710	0.326	−0.132
4. Recognize the importance of cultural background and lived experience of the person living with dementia when developing a plan of care	0.633	0.167	−0.077
5. Assist the caregiver in identifying available resources and services	0.608	−0.170	0.238
6. Communicate with a multidisciplinary team of health and social service providers when caring for a person living with dementia	0.514	0.104	0.175
7. Differentiate dementia from delirium or depression	0.472	0.097	0.181
8. Describe, in lay language, the progression of dementia	0.461	0.169	0.225
9. Analyze the ethical and legal parameters of providing care for a person living with dementia	0.374	0.314	0.165
10. Identify cognitive and noncognitive symptoms of dementia	0.339	0.137	0.333
11. Adapt verbal communication strategies to meet the needs of a person living with dementia	−0.069	1.062	0.092
12. Adapt nonverbal communication strategies to meet the needs of a person living with dementia	0.152	0.755	0.016
13. Identify signs of possible self-neglect, neglect, abuse, or exploitation in a person living with dementia	0.008	0.142	0.943
14. Identify signs of possible self-neglect, neglect, abuse, or exploitation in a caregiver providing care for a person living with dementia	0.103	−0.015	0.798
15. Identify ways to promote your personal safety when working with person living with dementia	0.110	0.398	0.447

* Promax rotation.

**Table 3:** Three Factor Dementia Care Competency Model.

Dementia Care Competency	Sub-Competencies
Global Dementia Knowledge	Integrate cultural background and lived experience into plan of care
	Incorporate support and resources into plan of care
	Recognize signs and symptoms of caregiver stress and burnout
	Distinguish the signs, symptoms, and progression of dementia
	Facilitate coordination and continuity of care with a multi-disciplinary team
Communication	Ability to facilitate discussion and education about legal and ethical concerns regarding ADRD
	Use of communication to promote patient centered care planning
	Situational adaptability of verbal and non-verbal communication
	Promotion of safety through effective communication
Safety	Recognition of the signs and symptoms of neglect, self-neglect, and abuse in the family/caregiver and/or those with ADRD
	Promotion of personal safety when delivering care

## Data Availability

The data are available from the corresponding author upon reasonable request.

## Appendix A

Dementia Care Competencies for Educational Programs Crosswalk.

**Table A1:** Articles used.

Dementia Care Competency Framework 2016	Dementia Care Practice Recommendations 2018	Dementia Management Quality Measurement Set Update 2018 Implementation Update	Competency Guide for Dementia Care: Direct Care Worker Workforce Development
*Dementia Care Competency Framework,* (2016). Agency for Integrated Care. https://www.aic.sg/partners/Documents/CMH%20Resources/Dementia%20Care%20Competency%20Framework.pdf [11 January 2022]	Fazio, et al. (2018). Alzheimer’s association dementia care practice recommendations. *Gerontologist, 58*(S1), S1–S9. https://doi.org/10.1093/geront/gnx182/ [11 January 2022]	*Dementia management* *quality measurement set update:* *2018 implementation update* (2018). American Medical Association, American Academy of Neurology Institute and American Psychiatric Association. https://www.aan.com/siteassets/home-page/policy-and-guidelines/quality/quality-measures/2018-dementia-management-measures.pdf [11 January 2022]	*Competency guide for dementia care: Direct care worker workforce development.* (2016). Georgia Alzheimer’s and Related Dementias Collaborative. https://aging.georgia.gov/sites/aging.georgia.gov/files/GARD%20Competency%20Guide_PDF.pdf [11 January 2022]

**Table A2:** Competencies Identified.

	Survey Rating Competency	Core Domain(s):	Goals	Quality Measure	Priority Training Topic
1	Describe, in lay language, the progression of dementia	Dementia Education - Basic Level	Detection and Diagnosis Transitions and coordination of services	Disclosure of Dementia Diagnosis Education and Support of Caregivers for Patients with Dementia	Understanding Dementia
2	Identify cognitive and non-cognitive symptoms of dementia	Dementia Education - Basic Level	Detection and Diagnosis Ongoing care for behavioral and psychological symptoms of dementia	Disclosure of Dementia Diagnosis	Understanding Dementia
3	Differentiate dementia from delirium or depression	Dementia Education - Basic Level	Detection and Diagnosis Medical Management	Disclosure of Dementia Diagnosis	Understanding Dementia
4	Adapt verbal communication strategies to meet the needs of a person living with dementia	Interacting with Persons with Dementia - Basic Level	Supportive and Therapeutic Environments Ongoing care for behavioral and psychological symptoms of dementia	Screening and Management of Behavioral and Psychiatric Symptoms Associated with Dementia	Communication
5	Adapt non-verbal communication strategies to meet the needs of a person living with dementia	Behaviors of Concern - Basic Level	Supportive and Therapeutic Environments Ongoing care for behavioral and psychological symptoms of dementia	Screening and Management of Behavioral and Psychiatric Symptoms Associated with Dementia	Communication
6	Develop a person- centered plan of care for person living with dementia	Person-Centered Care - Intermediate Level	Person-Centered Care Transitions and coordination of services Medical Management	Pain Assessment and Follow-up for Patients with Dementia Pharmacological Treatment of Dementia Education and Support of Caregivers for Patients with Dementia	Person-Centered Care Empowering the Person and Enriching Their Life
7	Recognize the importance of cultural background and lived experience of the person living with dementia when developing a plan of care	Person-Centered Care - Basic Level	Person-Centered Care Transitions and coordination of services Medical Management	Education and Support of Caregivers for Patients with Dementia Functional Status Assessment for Patients with Dementia	Person-Centered Care Empowering the Person and Enriching Their Life
8	Analyze the ethical and legal parameters of providing care for a person living with dementia	Palliative Care for Persons with Dementia - Intermediate Level Patient-Centered Care - Intermediate Level	Information, education, and support Medical Management	Functional Status Assessment & Driving Screening and Follow-up for Patients with Dementia Advance Care Planning and Palliative Care Counseling for Patients with Dementia	Reduction of Preventable Hospitalization Palliative and End of Life Care
9	Incorporate support for the family caregiver into the plan of care for a person living with dementia	Enriching Lives - Intermediate Level Patient-Centered Care - Intermediate Level	Information, education, and support Assessment and Care Planning	Education and Support for Caregivers for Patients with Dementia	Person-Centered Care Communication
10	Recognize signs and symptoms of caregiver stress and burnout	Care for Self and Caregivers - Basic Level Patient-Centered Care - Intermediate Level	Information, education, and support Assessment and Care Planning	Education and Support for Caregivers for Patients with Dementia	Person-Centered Care Reduction in Preventable Hospitalizations
11	Assist the caregiver in identifying available resources and services	Care for Self and Caregivers - Intermediate level Patient-Centered Care - Intermediate Level	Information, education, and support Assessment and Care Planning	Education and Support for Caregivers for Patients with Dementia	Person-Centered Care Reduction in Preventable Hospitalizations
12	Communicate with a multi-disciplinary team of health and social service providers when caring for a person living with dementia	Person Centered Care -Intermediate Level	Transitions and Coordination of Care Staffing Assessment and Care Planning	Education and Support for Caregivers for Patients with Dementia Advance Care Planning and Palliative Care Counseling for Patients with Dementia	Communication Reduction in Preventable Hospitalizations
13	Identify signs of possible self-neglect, neglect, abuse, or exploitation in a person living with dementia	Interacting with Persons with Dementia - Basic Level	Medical Management Assessment and Care Planning Person Centered Care	Safety Concern Screening and Follow-up for Patients with Dementia	Prevention and Reporting of Abuse Person- Centered Care
14	Identify signs of possible self-neglect, neglect, abuse, or exploitation, in a caregiver providing care for a person living with dementia	Care for Self and Caregivers - Basic Level	Information, education, and support Assessment and Care Planning	Education and Support for Caregivers for Patients with Dementia	Prevention and Reporting of Abuse
15	Identify ways to promote your personal safety when working with persons living with dementia	Care for Self and Caregivers - Basic Level	Supportive and Therapeutic Environments Person Centered Care	Safety Concern Screening and Follow-up for Patients with Dementia	Understanding Dementia Communication

## Appendix B

**Table A3:** Educator Survey with Dementia Competency Pairing.

Question Number	Question Content How Confident Are You That Graduates of your Program Have the Ability to					Dementia Care Competency
1	Describe, in lay language, the progression of dementia.					Competency 1: Knowledge of Dementia
2	Identify cognitive and non-cognitive symptoms of dementia.					Competency 1: Knowledge of Dementia
3	Differentiate dementia from delirium or depression.					Competency 1: Knowledge of Dementia
4	Adapt verbal communication strategies to meet the needs of a person living with dementia.					Competency 2: Communication /Interaction with Person with Dementia
5	Adapt non-verbal communication strategies to meet the needs of a person living with dementia.					Competency 2: Communication /Interaction with Person with Dementia
6	Develop a person-centered plan of care for persons living with dementia.					Competency 3: Person-Centered Care
7	Recognize the importance of cultural background and lived experience of the person living with dementia when developing a plan of care.					Competency 3: Person-Centered Care
8	Analyze the ethical and legal parameters of providing care for a person living with dementia.					Competency 3: Person-Centered Care
9	Incorporate support for the family caregiver into the plan of care for a person living with dementia.					Competency 4: Interdisciplinary Care
10	Assist the caregiver in identifying available resources and services.					Competency 4: Interdisciplinary Care
11	Communicate with a multi-disciplinary team of health and social service providers when caring for a person living with dementia					Competency 4: Interdisciplinary Care
12	Identify signs of possible self-neglect, neglect, abuse, or exploitation in a person living with dementia.					Competency 4: Interdisciplinary Care
13	Identify signs of possible self-neglect, neglect, abuse, or exploitation in a caregiver providing care for a person living with dementia.					Competency 4: Interdisciplinary Care
14	Identify ways to promote your personal safety when working with persons living with dementia.					Competency 5: Care for Self and Caregivers
15	Recognize signs and symptoms of caregiver stress and burnout					Competency 5: Care for Self and Caregivers
Available responses to above questions included the following:						
	Not Confident	Slightly Confident	Moderately Confident	Very Confident	Don’t Know	
